# Kinome profiling of BLV-induced ovine leukemia: an approach for identifying altered signaling pathways associated with oncogenesis

**DOI:** 10.1186/1742-4690-8-S1-A16

**Published:** 2011-06-06

**Authors:** Anne Van den Broeke, Ryan Arsenault, Nicolas Rosewick, Yvette Cleuter, Philippe Martiat, Arsène Burny, Scott Napper, Philip Griebel

**Affiliations:** 1 Laboratory of Experimental Hematology, Institut Jules Bordet, Université Libre de Bruxelles (ULB), 1000 Brussels, Belgium; 2 Pathogenomics, Vaccine and Infectious Disease Organization, University of Saskatchewan, Saskatoon, SK, Canada, S7N 5E3

## 

Transcriptome and miRnome information will likely be of significant value to our elucidation of the molecular mechanisms that govern cell transformation. However, an equally, if not more important goal, is to define those proteins that participate in signaling pathways that ultimately control cell fate.

Bovine Leukemia Virus (BLV), a delta-retrovirus closely related to HTLV-1, is associated with B-cell leukemia in sheep. We have employed kinome arrays which contain ovine peptide substrates selected to target known phosphorylation sites in proteins regulating key cell signaling pathways. Our data provide a quantitative measure of the phosphorylation activity of 300 kinases. We found significant changes in phosphorylation patterns of primary ovine leukemia/lymphoma versus normal B-cells. Pathway analysis tools revealed changes in proteins playing a major role in signaling cascades that determine cell-cycle entry, proliferation, survival and differentiation. Interestingly, analysis of cultured transformed B-cell lines suggested cell signaling events that characterize primary cancer cells were not conserved in vitro. Using NOD-Scid-Gamma immunodeficient mice and SC injection of ovine transformed B cell lines generated in vitro, we asked if transformed B-cells grown in mice would reflect the initial in vivo kinome profile identified in leukemic sheep. Finally, a priority identified for defining the rigour of our dataset was kinome analysis of an increased number of normal B-cells to provide an estimate of reference kinome diversity in an outbred population. Altogether, these investigations will provide a critical analysis of the utility of kinome arrays as a technology to analyze oncogenesis, identify therapeutic targets, and select potential cancer biomarkers.

